# Effects of Sources or Formulations of Vitamin K_3_ on Its Stability during Extrusion or Pelleting in Swine Feed

**DOI:** 10.3390/ani11030633

**Published:** 2021-02-27

**Authors:** Huakai Wang, Pan Yang, Longxian Li, Nan Zhang, Yongxi Ma

**Affiliations:** State Key Laboratory of Animal Nutrition, College of Animal Science and Technology, China Agricultural University, Beijing 100193, China; huakaiwhk@cau.edu.cn (H.W.); ypan23@163.com (P.Y.); S20193040586@cau.edu.cn (L.L.); S20203040629@cau.edu.cn (N.Z.)

**Keywords:** vitamin K_3_, retention, crystal, micro-capsule, micro-sphere, extrusion, pelleting

## Abstract

**Simple Summary:**

Pelleting or extrusion have been used in the feed industry to improve nutrient utilization and reduce the load of pathogenic microorganisms. High temperature and high pressure during this process cause loss of heat-sensitive vitamins, such as vitamin K_3_ (VK_3_). Encapsulation techniques have been gradually applied to feed additives, which may improve the stability of vitamins. Our previous work has shown that VK_3_ was the most unstable vitamin during heat processing. Therefore, the aims of this study were to determine the effects of sources, formulations of VK_3_, extrusion temperature and pelleting parameters on its stability. The results indicate that menadione nicotinamide bisulfite (MNB) is more stable than menadione sodium bisulfite (MSB) during high temperature and high-pressure processing. The micro-capsule/micro-sphere formulation VK_3_, manufactured through the encapsulation technique, had a higher recovery during pelleting but not during extrusion. Therefore, a reasonable decrease in the intensity of feed processing and choosing a more suitable formulation of VK_3_ could be a solution for accurate VK_3_ nutrition, according to current research results.

**Abstract:**

Two studies were conducted to determine the stability of vitamin K_3_ (VK_3_) in swine diets during extrusion or pelleting. The two sources were menadione sodium bisulfite (MSB) and menadione nicotinamide bisulfite (MNB), and the three formulations were crystal micro-capsule formulation and micro-sphere formulation. The recovery of six types of VK_3_ in swine diets was investigated after extrusion at 100 °C or 135 °C in Experiment 1. The recovery of six types of VK_3_ was investigated when the diets were pelleted at 60 °C (low temperature; LT) or 80 °C (high temperature; HT) and the length to diameter ratios were 5.2:1 (low length to diameter ratio; LR) or 7.2:1 (high length to diameter ratio; HR) in Experiment 2. In Experiment 1, MNB recovery (72.74%) was higher than MSB recovery (64.67%) after extrusion, while recovery of VK_3_ of crystal (74.16%) was higher than the recovery of micro-capsule (65.25%) and micro-sphere (66.72%). The recovery of VK_3_ (70.88%) was higher when extruded at 100 °C than that at 135 °C (66.54%). In Experiment 2, MNB recovery (86.21%) was higher than MSB recovery (75.49%) after pelleting, while the recovery of VK_3_ of micro-capsule (85.06%) was higher than the recovery of crystal (81.40%) and micro-sphere (76.09%). The recovery of VK_3_ (75.50%) was lower after HTHR pelleting than LTLR (83.62%), LTHR (81.52%) or HTLR (82.76%) treatment. Our results show that MNB has greater stability than MSB. VK_3_ of crystal or VK_3_ of micro-capsule were recommended for extrusion or pelleting, respectively.

## 1. Introduction

Extrusion or pelleting are the two main heat processing procedures in the feed industry, which can improve the digestibility of nutrients and the feed conversion rate of animals, reduce the contamination of feed by microorganisms, and extend the storage period of feed [1,2]. However, they have also contributed to a loss of vitamins [3]. Although over-fortification of vitamins in diets is often used to compensate for expected losses, this practice does not always ensure that animals have adequate and balanced micro-nutrients [4]. VK_3_ has the least stability, which is a good index to evaluate the stability of vitamins. Marchetti et al. [3] reported that micro-encapsulation can improve the stability of vitamins. In addition, preparation of micro-sphere has also been used to improve the stability or mask the odor of some nutrients [5,6], while there are few data on the effect of encapsulation on VK_3_ stability during feed processing. Therefore, the objective of this study was to determine the recovery of VK_3_ in swine diets after extrusion or pelleting and characterize the effects of source and formulation of VK_3_ on its stability during processing.

## 2. Materials and Methods

This study was conducted at the Ministry of Agriculture and Rural Affairs Feed Efficacy and Safety Evaluation Center located at China Agricultural University (Beijing, China). The extrusion of diets was conducted at the Institute of Food Science and Technology Chinese Academy of Agricultural Sciences (Beijing, China), and pelleting was completed at the China Agricultural University Feed Mill Educational Unit (Beijing, China).

### 2.1. Diets and Processing Parameters

Six types of VK_3_ were obtained from the Wellroad Animal Health Co. Ltd., China, as follows: menadione sodium bisulfite (MSB) of crystal, MSB of micro-capsule, MSB of micro-sphere, menadione nicotinamide bisulfite (MNB) of crystal, MNB of micro-capsule and MNB of micro-sphere. The analysis of VK_3_ in these six formulations is shown in Table S1. Six trace vitamin mineral (VTM) premixes were formulated using different VK_3_ formulations. The VTM premix composition is shown in Table S2. Six experimental diets (other ingredients are consistent, except the VTM premix) were designed to meet the nutritional requirement of piglets according to the Nutrient Requirements of Swine, and based on the corn and soybean diet [7]. The chemical compositions of experimental diets are shown in Table S3. Corn and soybean were ground according to methods described previously [8]. Processing parameters were consistent with the previous study except for extrusion temperature, pelleting temperature and length to diameter ratio (LD) [8].

### 2.2. Experiment 1

Experiment 1 was designed by a 2 × 3 × 2 factorial arrangement to evaluate the effects of two sources (MSB vs. MNB) and three formulations (crystal vs. micro-capsule vs. micro-sphere), and two extrusion temperatures (100 °C vs. 135 °C) on VK_3_ stability during extrusion. VTM premixes from each product were proportionally mixed into 400 kg of completed diets using a horizontal single ribbon mixer (Model SLHY 2.5c, Muyang, Yangzhou, China) for 240 s of dry mixing time. The mixed samples were used to measure initial VK_3_ content in the diet. Three batches of each of the six swine diets were extruded in a single screw annular gap extruder (DSE-25, Brabender Technologie GmbH & Co. KG, Essen, Germany) with screw diameter of 25 mm and LD of 20:1, differing in extrusion temperature (100 °C or 135 °C). The extruder was flushed and warmed with before proceeding with the experimental batches, and samples were collected after the extruder barrel temperature was stable. The individual batches were extruded from 100 °C to 135 °C and, before changing feed, the extruder was flushed with the experimental diets. Each batch represented one replicate and three sub-samples were obtained at evenly spaced intervals during the extrusion process in each batch.

### 2.3. Experiment 2

Experiment 2 was designed with a 2 × 3 × 4 factorial arrangement to evaluate the effects of two sources (MSB vs. MNB) and three formulations (crystal, micro-capsule or micro-sphere), and four pelleting parameters (condition temperatures and LD were 60 °C/5.2:1, 60 °C/7.2:1, 80 °C/5.2:1 and 80 °C/7.2:1) on VK_3_ stability in the feed. Two die configurations were 5.2:1 or 7.2:1 with dimensions of 2.5 × 13 mm or 2.5 × 18 mm. The mixed diets were prepared using the same procedure as in Section 2.2. The mixed samples were used to measure initial VK_3_ content in the diet. Diets were conditioned in a double-shaft steam conditioner (Famsun, SBTZ 10, Yangzhou, China) and then pelleted using a pellet mill (Famsun 180, Yangzhou, China). Three batches of each diet were pelleted and each batch represented one replicate. The pellet mill die was warmed with the diets before proceeding with the experimental batches. The individual batches were pelleted from 60 °C to 80 °C of conditioning temperature, pelleting the batch with the 5.2 of LD first, and before changing feed or transitioning to the 7.2 of LD the conditioner and pellet mill were flushed with the diets. Three sub-samples were obtained at evenly spaced intervals during the pelleting process in each batch.

The samples of extrusion or pelleting were dried and cooled in a custom manufactured 60 × 40 cm tray, which was placed in a vertical counter-flow custom-manufactured cooler, and cooled over open grates with circulating air. Samples were crushed with a grinder (FW-100, Beijing, China) before laboratory analysis.

### 2.4. Chemical Analyses of Ingredients

The VK_3_ and dry matter content of all feed ingredients were analyzed. The dry matter content of the sample was determined to calculate the VK_3_ recovery based on dry matter [9]. The amount of VK_3_ was detected by high performance liquid chromatography (HPLC) [10]. In brief, a 5 g sample was extracted with 50 mL of trichloromethane, 5 mL sodium carbonate solution and 5 g adsorbent were added and, after 30 min of rotating shock, the extract passed through a 0.45 μm filter membrane into the HPLC system, and the UV detection wavelength was 251 nm. The samples were assayed in triplicates. According to the previous study [8], we took the recovery of VK_3_ as its stability measurement index, and defined it as follows: the recovery of VK_3_(%) = nutrient content per gram of processed feed × feed weight (gram) after processing/(nutrient content per gram of raw feed × feed weight (gram) before processing) × 100. The VK_3_ concentration in unmanufactured mash diet is shown in Table S4.

### 2.5. Statistical Analysis

All data were analyzed using analysis of variance (ANOVA) with the Proc MIXED procedure of SAS (SAS Institute Inc., Carry, NC, USA). The statistical model included the fixed main effects of source, formulation, extrusion temperature, and their interaction effects in Experiment 1. The statistical model included the fixed main effects of source, formulation, pelleting parameter, and their interaction effects in Experiment 2. Batch was also included in the model as a random effect in Experiments 1 and 2. A single degree of freedom contrast was performed for the comparison between MSB and MNB, crystal, micro-capsule and micro-sphere, low temperature and high temperature, to validate the effect of source, formulation and extrusion temperature on VK_3_ recovery. A single degree of freedom contrast was performed for the comparison between MSB and MNB, crystal, micro-capsule and micro-sphere, LTLR, LTHR, HTLR and HTHR to validate the effect of source, formulation and pelleting parameter on VK_3_ recovery. Meanwhile, statistical differences among mean values were separated by Tukey’s multiple comparison [Tables S5 and S6]. Statistical significance was considered at *p* < 0.05.

## 3. Results

### 3.1. Effect of Source and Formulation of Vitamin K_3_ and Extrusion Temperature on Stability

The recovery of MNB (72.74%) was significantly higher than that of MSB (64.67%) (*p* < 0.05, Table 1). The recovery of VK_3_ of crystal (74.16%) was significantly higher than micro-capsule (65.25%) and micro-sphere (66.72%) (*p* < 0.05). The recovery of VK_3_ was significantly higher at low temperature (70.88%) than high temperature (66.54%) (*p* < 0.05). In addition, there were interaction effects between source and formulation, source and temperature, and formulation and temperature (*p* < 0.05), as the recovery of MNB of micro-capsule (62.48%) was lower than that of MSB of crystal (67.97%) at LT (*p* < 0.05, Table S5). Furthermore, the recovery of MNB of crystal (79.78%) at HT was higher than that of MSB at LT (67.97%) (*p* < 0.05). The recovery of MSB of micro-capsule (68.16%) at HT was higher than that of micro-sphere formulation (61.27%) at LT (*p* < 0.05).

### 3.2. Effect of Source and Formulation of Vitamin K_3_ and Pelleting Parameters on Its Stability

The recovery of MNB (86.21%) was significantly higher than MSB (75.49%) (*p* < 0.05, Table 2). The recovery of VK_3_ of micro-capsule (85.06%) was significantly higher than crystal (81.40%) and micro-sphere (76.09%) (*p* < 0.05). The recovery of VK_3_ was significantly lower after HTHR pelleting (75.50%) than after LTLR (83.62%), LTHR (81.52%) and HTLR (82.76%) pelleting (*p* < 0.05). Moreover, there were interaction effects between source and formulation, source and processing, formulation and processing, source, and formulation and processing (*p* < 0.05), as the recovery of MNB of micro-sphere (73.18%) was lower than that of MSB of micro-capsule (82.35%) at HTLR (*p* < 0.05, Table S6). There was no statistical difference between the recovery of MNB of crystal (84.59%) at LTLR and the recovery of MSB of crystal (83.03%) at HTLR. Meanwhile, the recovery of MNB of micro-capsule (91.54%) at HTHR was higher than that of micro-sphere formulation (84.59%) at LTLR (*p* < 0.05).

## 4. Discussion

Our previous work [8] reported that microencapsulated vitamins had greater stability compared to non-microencapsulated vitamins during extrusion and pelleting, and VK_3_ was the most unstable vitamin. In the previous study, we coated all the vitamins together, as it is not clear whether coating VK_3_ alone can effectively improve its stability. Moreover, whether the micro-sphere formulation can improve the stability of vitamins is also worth studying. Therefore, we selected the VK_3_ of two sources to explore the effects of micro-capsule and micro-sphere on stability during extrusion or pelleting.

### 4.1. Effect of Vitamin K_3_ Source on its Stability

The VK_3_ in the formulation of MSB and MNB is intended for use and recommended to be incorporated in compound feed via premixes. Coelho [11] reported that MNB was more stable than MSB during the pelleting process, which is consistent with our results. This may be attributed to the molecular structure of MNB. The MNB was synthesized from MSB by introducing nicotinamide macromolecules to replace sodium ions and 3 crystalline water in MSB [12]. However, the European Food Safety Authority [12] reported that pelleting reduced MSB of crystal to about 53% of the initial content (pelleting at 90 °C, 6 mg MSB/kg feed) and reduced MNB of crystal by about 52% of the initial content (pelleting at 70 °C, 4.5 mg MNB/kg feed), which is different to our results. One reason for this disagreement may be because of the improvement of current vitamin processing technology. Moreover, the content of VK_3_ in diet may also be an influence. Formulation and processing had significant interaction effects with source of VK_3_, which may result in MSB recovery being higher than MNB in some situations. Therefore, our results indicate that MNB has a higher stability than MSB, while formulation and different processing methods also need to be considered in practical applications.

### 4.2. Effect of Vitamin K_3_ Formulation on its Stability

Traditionally, vitamins are added in the formulation of crystal, but their potency is greatly reduced after processing or storage, meaning that its activity cannot be guaranteed. It is believed that micro-capsule and micro-sphere technology can extend the shelf-life and improve the bioavailability of vitamins [13]. The principle is mainly based on reducing the contact of the active ingredients with the adverse environment [14]. Marchetti et al. [3] conducted an experiment to investigate the stability of crystalline or fat-coated vitamins after the extrusion or pelleting process of fish feeds, and found that the coated VK_3_ was more stable than the crystalline VK_3_ whether in extrusion or pelleting. Our data show that micro-capsule can improve the recovery of VK_3_ only during pelleting. However, the results of our former study did not show that micro-capsule or micro-sphere can improve the stability of VK_3_ [8]. This may be due to the difference of encapsulating material, the micro-capsule technology or the improvement of pelleting technology [13,15]. Additionally, the source and processing methods may also influence the effects of micro-capsule and micro-sphere. In order to effectively improve VK_3_ stability, appropriate encapsulating materials should be researched to ensure better results.

### 4.3. Effect of Extrusion Temperature on the Stability of Vitamin K_3_

Extrusion is a thermal denaturation process via a combination of moisture, pressure, temperature and mechanical shear [16]. The extrusion temperature is above 90 °C under normal production conditions. This high temperature is used to improve digestibility through gelatinization of starch, denaturation of proteins and inactivation of anti-nutritional factors [8,16]. Previous results [17] showed that 20% of VK_3_ was destroyed when the extrusion temperature was 93 °C, but 70% of VK_3_ was destroyed when the extrusion temperature was 149 °C, which is consistent with the present data. This may be due to the fact that high temperature will accelerate the oxidant reaction and destroy the internal structure of the VK_3_ molecule [17]. Additionally, the recovery of VK_3_ decreased with the increase of screw speed, decrease of water content and increase of energy input during extrusion [18].

### 4.4. Effect of Pelleting Process on the Stability of Vitamin K_3_

Friction, pressure, temperature, humidity, and conditioning time are major factors affecting the stability of vitamins during the pelleting process [19]. The recovery of VK_3_ decreased with the extension of conditioning time, increase of LD and increase of friction. Higher LD decreased the VK_3_ recovery at high temperature, which agreed with the previous study [9]. Higher LD increases the friction and temperature between feeds. The damaging effect of high temperature is mainly due to: (1) VK_3_ has a low melting point and is susceptible to high temperature, and (2) high temperature provides energy for redox reaction and accelerates the destruction of VK_3_.

## 5. Conclusions

Extrusion or pelleting significantly reduce the recovery of VK_3_. We suggest that MNB should be recommended as the VK_3_ source, the micro-capsule formulation should be recommended during pelleting, and the crystal formulation should be recommended during extrusion. Furthermore, the temperature and LD should be reduced as much as possible during thermal processing of feed to reduce the loss of VK_3_ potency. Development of micro-capsule and micro-sphere carriers and further technical updates are needed to improve vitamin stability.

## Figures and Tables

**Table 1:** Effects of sources and formulations of vitamin K_3_ and extrusion temperature on its stability.

	Source (A)		Formulation (B)			Temperature (C)			*p*-Values						
	MSB	MNB	Cr	Mic-c	Mic-s	LT	HT	SEM	A	B	C	A × B	A × C	B × C	A × B × C
Sample size (n)	54	54	36	36	36	54	54								
VK_3_ recovery (%)	64.67 ^B^	72.74 ^A^	74.16 ^a^	65.25 ^b^	66.72 ^b^	70.88 ^α^	66.54 ^β^	0.81	<0.001	<0.001	<0.001	<0.001	<0.001	<0.001	0.307

^A,B^ Means in a row, with different superscripts, are different (*p* < 0.05) under source. ^a,b^ Means in a row, with different superscripts, are different (*p* < 0.05) under formulation. ^α,β^ Means in a row, with different superscripts, are different (*p* < 0.05) under temperature. MSB, menadione sodium bisulfite; MNB, menadione nicotinamide bisulfite; Cr, crystal; Mic-c, micro-capsule; Mic-s, micro-sphere; LT, low temperature (100 °C); HT, high temperature 7(135 °C); VK_3_, vitamin K_3_; SEM, Standard Error of Mean; A, Source; B, Formulation; C, Temperature.

**Table 2:** Effects of sources and formulations of vitamin K_3_ and pelleting parameter on its stability.

	Source (A)		Formulation (B)			Processing (C)					*p*-Values						
	MSB	MNB	Cr	Mic-c	Mic-s	LTLR	LTHR	HTLR	HTHR	SEM	A	B	C	A × B	A × C	B × C	A × B × C
Sample size (n)	108	108	72	72	72	54	54	54	54								
VK_3_ recovery (%)	75.49 ^B^	86.21 ^A^	81.40 ^b^	85.06 ^a^	76.09 ^c^	83.62 ^α^	81.52 ^α^	82.76 ^α^	75.50 ^β^	0.56	<0.001	<0.001	<0.001	<0.001	<0.001	<0.001	<0.001

^A,B^ Means in a row, with different superscripts, are different (*p* < 0.05) under source. ^a,b,c^ Means in a row, with different superscripts, are different (*p* < 0.05) under formulation. ^α,β^ Means in a row, with different superscripts, are different (*p* < 0.05) under process. MSB, menadione sodium bisulfite; MNB, menadione nicotinamide bisulfite; Cr, crystal; Mic-c, micro-capsule; Mic-s, micro-sphere; LD, length to diameter ratio; LTLR, low temperature + low LD (60 °C/5.2:1); LTHR, low temperature + high LD (60 °C/7.2:1); HTLR, high temperature + low LD (80 °C/5.2:1); HTHR, high temperature + high LD (80 °C/7.2:1); VK_3_, vitamin K_3_; SEM, Standard Error of Mean; A, Source; B, Formulation; C, Temperature.

## Data Availability

Not applicable.

## Supplementary Materials

The following are available online at https://www.mdpi.com/2076-2615/11/3/633/s1, Table S1: The analysis of VK_3_ in these six formulations, Table S2: Composition of vitamin mineral premix, Table S3: Ingredient composition and calculated nutrient composition of experimental diets (g/kg, as-fed basis), Table S4: Vitamin K_3_ concentration in unmanufactured mash diet (as-fed basis).Table S5: Effects of sources and formulations of vitamin K_3_ on its stability during extrusion, Table S6: Effects of sources and formulations of vitamin K_3_ on its stability during pelleting.
