# Supplementary material for: Effects of Sources or Formulations of Vitamin K3 on Its Stability during Extrusion or Pelleting in Swine Feed
Source: Animals (Basel). 2021 Feb 27;11(3):633. doi: 10.3390/ani11030633 (PMC7997351; doi:10.3390/ani11030633)
Supplement: Supplementary file 1 [file animals-11-00633-s001.pdf]

Supplementary Material Table S1: The analysis of VK_3 in these six formulations.

Vitamin K_3	Analyzed vitamin K_3 values (%)
MSB of crystal	50.00
MSB of micro-capsule	8.90
MSB of micro-sphere	6.25
MNB of crystal	43.90
MNB of micro-capsule	8.60
MNB of micro-sphere	5.94

Six types of VK_3 were provided by the Wellroad Animal Health Co. Ltd., China. The preparation materials of micro-capsule formulation mainly included stearic acid and glycerate. The preparation materials of micro-sphere formulation mainly included carboxymethyl cellulose and ethyl cellulose. MSB, menadione sodium bisulfite; MNB, menadione nicotinamide bisulfite.

Supplementary Material Table S2: Composition of vitamin trace mineral premix.

Item	premix (g/kg)	premix(g/kg)	premix(g/kg)	premix (g/kg)	premix(g/kg)	premix(g/kg)
Other vitamins	49.66	49.66	49.66	49.66	49.66	49.66
MSB of crystal	2.00					
MSB of micro-capsule		11.25				
MSB of mic-sphere			16.02			
MNB of crystal				2.28		
MNB of micro-capsule					11.64	
MNB of micro-sphere						16.85
Trace mineral	50.26	50.26	50.26	50.26	50.26	50.26

Other vitamins provided the following per kilogram of vitamin trace mineral premix: Vitamin A (retinol), 4,800 mg; Vitamin D_3 (cholecalciferol), 1,600mg; Vitamin E (D, L-α-tocopherol acetate), 40,000 mg; Vitamin B_1 (thiamine), 800 mg; Vitamin B_2 (riboflavin), 2,000 mg; Vitamin B_6 (pyridoxine hydrochloride), 1,200 mg; Vitamin B_{12} (cyanocobalamin), 10 mg; Niacin (nicotinic acid),11,000 mg; Pantothenic acid (D-pantothenic acid), 6,000 mg; Vitamin B_7 (biotin), 200 mg; Vitamin B_9 (folic acid), 50 mg. Trace mineral provided the following per kilogram of vitamin trace mineral premix: Cu ($CuSO_4$), 4000 mg; Fe ($FeSO_4$), 20,000 mg; Zn (ZnO), 20,000 mg; Mn (MnO), 6,000 mg; I [$Ca(IO_3)_2$], 200 mg; Se (NaSeO2), 60 mg. MSB, menadione sodium bisulfite; MNB, menadione nicotinamide bisulfite.

Supplementary Material Table S3: Ingredient composition and calculated nutrient composition of experimental diets (%, as-fed basis) [1].

Ingredient		Calculated nutrient values	
Corn	65.60	Digestible energy, MJ/kg	13.57
Soybean meal	21.05	Crude protein	16.87
Soybean oil	2.39	Calcium	0.77
Fish meal	1.91	Available phosphorus	0.38
Whey powder	0.96	Lysine	1.29
Monocalcium phosphate	0.84	Methionine	0.37
Limestone	0.91	Threonine	0.76
Salt	0.29	Tryptophan	0.21
Choline	0.10		
L-lysine HCl	0.70		
DL-methionine	0.14		

L-threonine	0.26
Tryptophan	0.07
VTM premix	4.78
Total	100.00

[1] Six experiment diets (Other ingredients are consistent except the VK_3 source) were designed to meet the nutritional requirement of piglets according to the Nutrient Requirements of Swine and based on the corn and soybean meal diet.

Supplementary Material Table S4: Vitamin K_3 concentration in unmanufactured mash diet (as-fed basis) [1].

Item	Source	Formulation	Calculated (mg/kg) [2]	Analyzed (mg/kg) [3]	Ratio (%) [4]	SD
Diet 1	MSB	crystal	47.80	46.69	97.68	2.19
Diet 2	MSB	micro-capsule	47.86	46.66	97.49	2.78
Diet 3	MSB	micro-sphere	47.86	45.65	95.38	1.24
Diet 4	MNB	crystal	47.84	45.74	95.61	1.55
Diet 5	MNB	micro-capsule	47.85	45.88	95.88	2.22
Diet 6	MNB	micro-sphere	47.84	48.70	101.80	2.22

[1] The experiment diets were formulated using different vitamin mineral (VTM) premix. [2] Calculated values were determined according to the composition of the diet. [3] Value represent the mean value of each repeated analysis sample. [4] Analyzed / calculated ratio. MNB, menadione nicotinamide bisulfite; T-H, temperature and relative humidity. SD, standard deviation.

Supplementary Material Table S5: Effects of sources and formulations of vitamin K_3 on its stability during extrusion.

Source	Formulation	Temperature	Vitamin K_3 recovery (%)
MSB	Crystal	LT	67.97^{cd}
		HT	63.40^{de}
	Micro-capsule	LT	70.06^{c}
		HT	68.16^{c}
	Micro-sphere	LT	61.27^{ef}
		HT	57.19^{f}
MNB	Crystal	LT	85.48^{a}
		HT	79.78^{b}
	Micro-capsule	LT	62.48^{e}
		HT	60.31^{ef}
	Micro-sphere	LT	78.04^{b}
		HT	70.37^{c}

[a, b, c, d, e, f] Means in a column, with different superscripts, are different ($P < 0.05$). MSB, menadione sodium bisulfite; MNB, menadione nicotinamide bisulfite; LT, low temperature (100°C); HT, high temperature (135℃).

Supplementary Material Table S6: Effects of sources and formulations of vitamin K_3 on its stability during pelleting

Source	Formulation	Processing	Vitamin K_3 recovery (%)
		Temperature/LD	
MSB	Crystal	LTLR	80.84^{fghi}
		LTHR	78.12^{i}

		HTLR	83.03^{efg}
		HTHR	71.98^{k}
	Micro-capsule	LTLR	79.97^{ghi}
		LTHR	73.27^{jk}
		HTLR	82.35^{fgh}
		HTHR	69.20^{k}
	Micro-sphere	LTLR	73.13^{k}
		LTHR	63.32^{l}
		HTLR	78.88^{hi}
		HTHR	71.99^{k}
MNB	Crystal	LTLR	84.59^{ef}
		LTHR	90.86^{cd}
		HTLR	84.12^{efg}
		HTHR	77.68^{ij}
	Micro-capsule	LTLR	96.01^{a}
		LTHR	93.30^{abc}
		HTLR	95.00^{ab}
		HTHR	91.54^{bcd}
	Micro-sphere	LTLR	87.17^{de}
		LTHR	90.23^{cd}
		HTLR	73.18^{k}
		HTHR	70.78^{k}

$^{a, b, c, d, e, f, g, h, i, j, k, l}$ Means in a column, with different superscripts, are different ($P < 0.05$). MSB, menadione sodium bisulfite; MNB, menadione nicotinamide bisulfite; LD, length to diameter ratio; LTLR, low temperature + low LD (60℃/5.2:1); LTHR, low temperature + high LD (60℃/7.2:1); HTLR, high temperature + low LD (80℃/5.2:1); HTHR, high temperature + high LD (80℃/7.2:1).
